# The Influence of Orthogeriatric Co-Management on Economical Outcomes After Treatment of Proximal Femoral Fractures: Real-World Data of Comparable Cohorts Originating from the Same Geographic Area

**DOI:** 10.3390/jcm14124149

**Published:** 2025-06-11

**Authors:** Samuel Känel, Manuel Känel, Method Kabelitz, Kim Aggeler, Michael Dietrich

**Affiliations:** 1 Department of Health Sciences and Technology, Eidgenössische Technische Hochschule Zürich, 8092 Zurich, Switzerland; skaenel@student.ethz.ch (S.K.); kaenelm@student.ethz.ch (M.K.); 2 Clinic for Orthopaedics, Hand- and Trauma Surgery, Stadtspital Zürich, 8037 Zurich, Switzerland; kim.aggeler@stadtspital.ch (K.A.); michael.dietrich@stadtspital.ch (M.D.)

**Keywords:** multidisciplinary care team, standard of care, orthopaedics, geriatric care, proximal femoral fractures, healthcare costs

## Abstract

**Background:** The global number of operatively treated proximal femoral fractures is steadily growing, driven by the demographic shift toward an increasingly elderly, frail, and comorbid population. This clinical condition profoundly impacts not only patient health but also the finances of healthcare systems. The aim of this economic analysis was to investigate the impact on direct costs of orthogeriatric co-management (OGCM) compared to standard of care (SOC). **Methods:** A retrospective analysis was conducted investigating two comparable cohorts of patients aged 75 and above, originating from the exact same geographic area, who underwent surgical treatment for proximal femoral fractures in 2023. The two cohorts differed in their perioperative care protocols: one followed an OGCM (*n* = 143) protocol, while the other adhered to a SOC protocol (*n* = 141). Economic data were retrieved from the centralised finance department managing the two hospital sites under investigation. **Results:** The findings revealed that the OGCM protocol was associated with direct costs that were not higher (CHF 16,019 vs. CHF 16,713, *p* = 0.78) compared to SOC, despite higher daily costs in the OGCM cohort (CHF 2504 vs. CHF 2202, *p* < 0.0001). This difference was largely driven by a significantly shorter length of stay in the OGCM group (6 days vs. 7 days, *p* = 0.002). **Conclusions:** Optimising resource allocation through tailored geriatric care protocols suggests not only an improvement in clinical outcomes but also a reduction in economic burden, thereby alleviating pressure on the healthcare system.

## 1. Background

Proximal femoral fractures are a major healthcare concern for the elderly population and the entire healthcare system [1,2,3]. Considering the demographic shift toward an increasingly older society, a growing number of proximal femoral fractures is expected, with global estimates predicting an increase to 6.26 million in 2050 [1,4,5]. Despite advances in healthcare, the one-year mortality rate continuously ranges between 20 and 30%, affecting approximately 18% of the female population and 6% of the male population worldwide [6,7,8]. Moreover, the presence of comorbidities, polypharmacy, and other disabilities must be taken into account, further complicating the treatment of geriatric patients [9,10,11]. Additionally, one should consider the financial burden associated with those fractures, showing an unceasing increase, with an estimated lifetime cost in the United States of US dollars (USD) 62 billion by 2040 [12]. The long-term consequences of this lifetime-changing event must also be kept in mind, with high costs associated with rehabilitation, home care, and readmissions often caused by complications [13,14]. Furthermore, increasing challenges must be addressed by the political decision-makers, having to plan and structure the healthcare system in a context where costs are continuously rising, driven by technical progress and the increase in the number of procedures [15]. Clinical and economic pressures have driven the development of a more efficient healthcare system, leading to orthogeriatric care protocols that not only consider surgical challenges but take also into account the medical needs of frail geriatric patients [16,17]. The differentiation between this type of care approach and standard protocols is mainly represented through the decision-making professionals responsible for the patient [1]. Orthogeriatric co-management (OGCM) protocols were first implemented starting from the 1950s in the United Kingdom, evolving over time with the aim of applying comprehensive treatments in a multidisciplinary setting on clinically complex patients [18,19]. Several studies have investigated OGCM protocols for the treatment of proximal femoral fractures, showing that a multidisciplinary, more personnel- and cost-intensive approach can be cost-effective [20,21,22]. When compared to a standard-of-care (SOC) protocol, lower mortality rates at 30 days and 1 year post-surgery and lower total costs can be achieved [14,21]. With specific setups, OGCM care protocols have been able to generate financial savings for hospitals through a more efficient allocation of available resources [1,23,24].

Although recent literature presents studies comparing OGCM with SOC protocols, no studies with well-established rules simultaneously applied on two cohorts of comparable size in the same period of time, with highly comparable patient cohorts from the same metropolitan area, have been identified. Furthermore, there is a lack of studies that had access to the individual components that constitute direct costs, in which highly comparable cohorts were analysed. Specifically, there are no studies comparing two treatment protocols, with patients exposed to the same risk factors, as well as the same environmental and socioeconomic factors, on a daily basis.

The aim of this study was to evaluate what impact an OGCM treatment pathway could have at a financial level compared to a SOC protocol, by analysing the direct costs generated by treating geriatric patients with proximal femoral fractures. Secondly, the study investigated whether the individual components of direct costs differed between the OGCM and SOC protocols.

## 2. Methods

### 2.1. Study Design

A retrospective single-centre cohort study was conducted. Demographic data were retrieved from the local electronic database (KISIM, Cistec AG, Zürich, Switzerland). All patients aged 80 years or above 75 years with two major secondary diagnoses, presenting a proximal femoral fracture between 1 January and 31 December 2023, were consecutively enrolled and retrospectively analysed. Inclusion criteria were an operatively treated proximal femoral fracture (trochanteric or femoral neck) after the occurrence of a low-energy trauma. Furthermore, a full set of economic data and a signed informed consent form had to be present. Patients with signs of atypical or pathological fractures and missing or unrepresentative data on direct costs were excluded. The study was reviewed and approved by the local Ethics Committee (BASEC number 2024-01660).

The analysed data were generated at two different sites within the same metropolitan area, whereas one site was certified for OGCM. At one location, an OGCM protocol was followed, and at the other location, a SOC treatment was conducted. The observed hospital centres were five kilometres apart and were centrally managed by a single institution. The included patients were treated by one surgical team active at both sites, using similar medical devices and operative techniques.

### 2.2. Orthogeriatric Co-Management

The OGCM care protocol has been applied to all patients admitted at Facility A since 2012, which was certified for the first time in 2018. All OGCM cases were treated in an orthogeriatric ward, created to take into account the specific needs of geriatric patients. The OGCM is a patient-oriented multidisciplinary care approach, characterised by a standardised care protocol from admission to discharge, in which the surgeon and geriatrician have shared responsibility for the care of individual cases. Each patient was followed in a standardised manner not only by the orthopaedic surgeon but also by the geriatrician, physiotherapist, nurses, nutritionist, social services, and other specialists. The medical course of patients was analysed twice a week in a multidisciplinary setting, making decisions by considering all parties involved in the care process. The involved geriatrician enabled the management of complex medical conditions present in elderly patients, by handling comorbidities and polypharmacy, preparing the patient optimally for surgery, reducing postoperative complications, and enhancing recovery. Discharge planning, carried out by social services, was initiated from the early stages of hospitalisation to ensure the best postoperative course suited to the patient’s condition. Physiotherapy sessions were performed twice every day, speeding the recovery of mobility from the earliest stages after surgery. The rationale behind this approach lies in the principles of geriatric care, where early and intensive mobilisation plays a crucial role in restoring functional independence. Increasing the frequency of physiotherapy supports earlier discharge, reduces the risk of complications related to immobility, and helps patients return more quickly to their usual living environment. This intensified rehabilitation strategy reflects the proactive and multidisciplinary nature of the OGCM model in addressing the complex needs of older adults after surgery.

### 2.3. Standard of Care

The SOC protocol was applied to all patients treated at Facility B, with the orthopaedic surgeon responsible for making decisions accordingly and determining the need for consultation with additional specialists, such as a geriatrician or nutritionist, if required. At this site, patients were admitted to the general ward without distinctions regarding diagnosis or age. The SOC protocol included a single physiotherapy session per day.

### 2.4. Data Source

The analysed economic data were retrieved from the finance department of the hospital, which calculated the costs of the individual procedures received by the patient during hospitalisation. The costs of the individual procedures were calculated based on REKOLE, the Swiss standard for operational hospital accounting, where it is specified how to calculate each individual cost.

### 2.5. Outcome Measures

Direct costs were the expenses generated by patients during their hospital stay. The costs generated in the two hospital centres were adjusted by standardising the cost of the tariffs for each performance, resulting in comparable expenses for the two protocols. All costs were calculated in Swiss francs (CHF) based on the price index of 2023, with an indicative exchange rate of 1 CHF = 1.14 USD. The most relevant components of direct costs included the following shares: drugs, blood and blood products, implants, patient surveillance, operating room, anaesthesia, intensive care unit (ICU), imaging procedures, laboratory tests, physiotherapy, nutrition counselling, social services, and room costs. Patient surveillance costs were generated by unstable patients during their hospital stay, defined based on clinical judgment as requiring increased monitoring due to factors such as hemodynamic or respiratory instability, acute cognitive fluctuations (e.g., delirium), or uncontrolled comorbidities. These cases demanded more frequent vital checks, closer observation, and increased nursing workload, contributing to higher surveillance-related expenses.

### 2.6. Statistical Analysis

All continuous variables were tested for normality distribution using the Shapiro–Wilk test, before being tested statistically. A non-parametric test was used to identify differences between the cohorts, using the Wilcoxon signed-rank test. Continuous variables were described with the median and interquartile range (IQR). For patient surveillance, costs were described with mean and standard deviation. Binary and categorical variables were tested with the Chi-square test or the Fisher exact test depending on the number of observations per category. These variables were described with absolute values and percentages. The significance level was set at *p* < 0.05. R software version 4.2.3 (R Core Team, Vienna, Austria) was used for the statistical analysis.

## 3. Results

**Figure 1:**
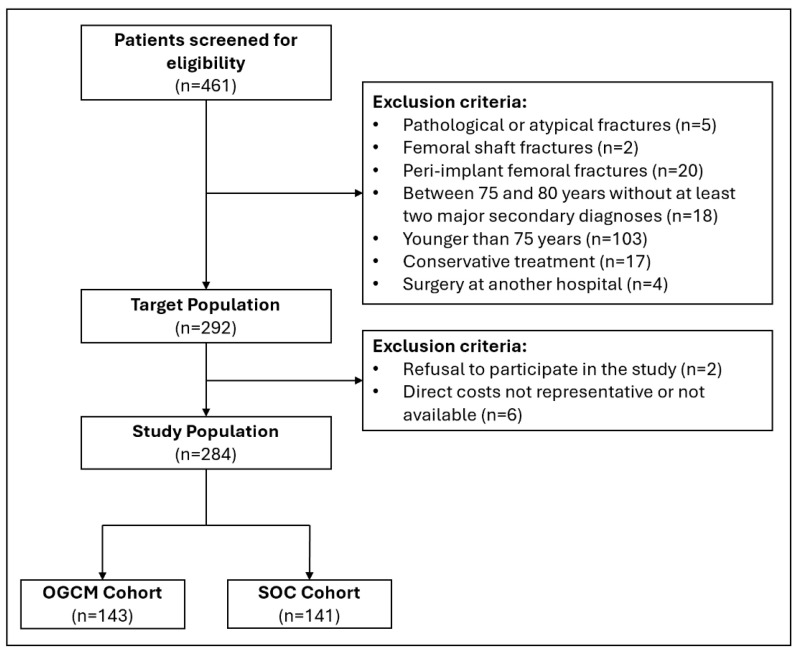
Flowchart of the patient inclusion process. SOC = standard of care. OGCM = orthogeriatric co-management.

After the screening for eligibility and application of the inclusion criteria, 143 patients were included for the OGCM protocol and 141 patients for the SOC protocol (Figure 1). The mean age of the patients in the OGCM cohort was 87.3 ± 5.6 years compared to 86.6 ± 6.2 years for the SOC (*p* = 0.25). Further basic demographic characteristics are reported in Table 1.

**Figure 2:**
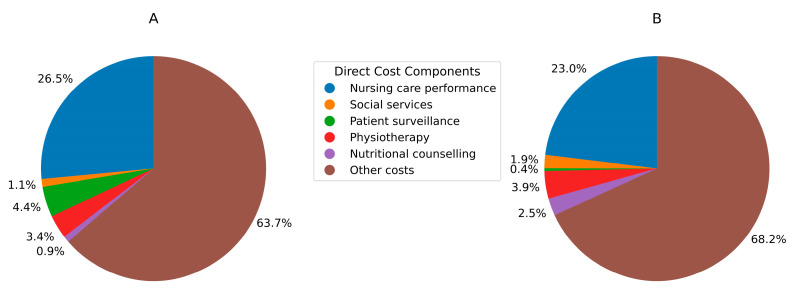
Proportions of the direct cost components analysed for the SOC (**A**) and OGCM (**B**) cohorts. Other costs included the direct cost components reported in the Materials and Methods section. SOC = standard of care. OGCM = orthogeriatric co-management.

The median direct cost was CHF 16,019 (IQR 14,223–19,560) for the OGCM protocol and CHF 16,713 (IQR 13,210–20,279) for the SOC treatment regime, which was not statistically significant (*p* = 0.78). Considering the median daily direct costs, the OGCM protocol generated CHF 2504 (IQR 2071–3407), and the SOC generated CHF 2202 (IQR 1829–2931), a difference that was statistically significant (*p* < 0.0001). Comparing daily costs, the OGCM protocol was 12.07% more expensive, resulting in a daily difference of CHF 302 per patient. The discrepancy in direct costs on an annual basis between the two compared protocols was CHF 97,854 if the 141 patients included in the SOC cohort were considered. Figure 2 presents the distribution of direct costs for each cohort.

Over the course of the hospital stay, the median nursing care costs were CHF 3786 (IQR 2546–5280) for the OGCM and CHF 3825 (IQR 2763–5808) following the SOC treatment regime (*p* = 0.21). Comparing the median costs of daily nursing care per patient, CHF 599 (IQR 490–719) was generated for OGCM compared to CHF 576 (IQR 454–663) with a SOC protocol (*p* = 0.15). The average cost for patient surveillance showed a significant difference between the two protocols, with respective costs of CHF 77 ± 451 for the OGCM and CHF 809 ± 1661 for the SOC (*p* < 0.0001). The average daily patient surveillance cost indicated a significant difference between the two protocols, resulting in lower expenses equivalent to CHF 14 (±91) following an OGCM protocol compared to CHF 108 (±228) for the SOC (*p* < 0.0001). Patients following the OGCM protocol incurred higher physiotherapy costs during hospitalisation, generating a median cost of CHF 568 (IQR 410–1151) compared to CHF 457 (IQR 297–913) for the SOC treatment model, resulting in a significant difference of 19.52% (*p* = 0.004). Simultaneously, the median daily cost of physiotherapy sessions in the OGCM protocol was significantly higher, with a respective cost of CHF 73 (IQR 59–83) in contrast to CHF 54 (IQR 42–63) for the SOC (*p* < 0.0001). Based on the various infrastructures, the median difference in the cost of nutrition counselling per patient throughout the hospitalisation period was statistically significant, generating an expenditure of CHF 422 (IQR 59–83) in the OGCM cohort and CHF 0 (IQR 0–324) in the SOC one (*p* < 0.0001). The comparison of the median daily nutrition counselling cost also showed a significantly higher cost in the OGCM cohort, generating CHF 51 (IQR 39–60) compared to CHF 0 (IQR 0–19) in the SOC cohort (*p* < 0.0001). Looking at the median cost of social services, it was CHF 328 (IQR 15–576) for the OGCM and CHF 31 (IQR 0–292) for the SOC, corresponding to a significantly higher cost of 90.65% using the OGCM protocol (*p* < 0.0001).

## 4. Discussion

Proximal femoral fractures have shown to be an ongoing challenge for the global healthcare system, with a huge impact on the financial resources of all stakeholders involved in the healthcare system [12]. In our study, an OGCM protocol did not incur higher costs compared to the SOC treatment regimen, despite adopting a more resource-intensive approach, both in financial and personnel terms.

Our analysis indicated that the multidisciplinary approach offered an efficient use of resources, providing more specialist interventions without resulting in increased costs compared to standard care. Two recent studies showed a significant reduction in hospital costs by means of OGCM, and the difference between the two protocols, which was 4.3% in our study, was 26.4% and 33.3% in the respective research groups [12,14]. Other researchers have reported higher overall hospital costs, dictated by national healthcare systems characterised by a different functioning of hospital cost reimbursement, where the implementation of OGCM protocols requires a minimal length of hospital stay (LOS) [8,13,17]. Several studies in the literature have attributed a substantial weight in the generation of hospital costs to the LOS, highlighting the central role of the geriatrician in reducing in-hospital complications, which negatively affect both the LOS and direct costs [22,25,26]. Confirming the above, a shorter LOS was also found in our study with the OGCM, with a median of 6 days compared to 7 days for the SOC (*p* = 0.002), pointing to the importance of involving several specialists in the treatment of geriatric patients.

The cost of nursing care was found to be comparable across both protocols when considering the costs generated during the entire period of hospitalisation. Regarding the daily cost of nursing care, the multidisciplinary approach did not differ significantly from the SOC but generated slightly higher expenses. As highlighted by Titler et al., an increased presence of nursing care contributes to a reduction in postoperative complications (Table 2), thus shortening the LOS, with a positive impact on the generated costs [15]. Analysing nursing costs in relation to complications, we observed that patients with delirium from the SOC cohort generated higher nursing costs than patients treated in a multidisciplinary manner, with a significant median difference of CHF 858 (*p* = 0.037). In addition, it must be considered that 52% of SOC delusional patients were in need of patient surveillance, compared to only 9% of OGCM patients, resulting in a significantly higher mean patient surveillance cost of CHF 773 with the SOC (*p* < 0.0001). Regarding these results, it can be inferred that the prevention of delirium should be a goal in the studied population, as it is a frequent complication and a source of additional direct costs for the affected patients [18,27,28]. When considering the entire study population, we similarly observed a significantly higher mean cost of patient surveillance with the SOC protocol. Unfortunately, no comparative studies have been identified, although the literature highlights the positive influence of the OGCM in preparing the patient for surgery by preventing the occurrence of postoperative complications, thus reducing costs [6,29]. It must be pointed out that patient surveillance was a procedure implemented in unstable patients post-surgery, being a symptomatic treatment of preoperatively often targetable and preventable complications. Considering that only 3% of the OGCM patients received patient surveillance, compared to 29% of the SOC patients (*p* < 0.0001), it is possible to objectively capture the benefits associated with structured care for complex patients based on standard order sets at the level of clinical outcomes.

Comparing physiotherapy expenses, both the total costs generated throughout the entire hospital stay and the daily costs were significantly higher under the OGCM protocol. Our findings align with those of Kimmel et al., where a more intense physiotherapy program throughout the hospital stay, potentially speeding up functional recovery, led to earlier patient discharge and lower direct costs [30].

With regard to the nutritional counselling costs, the significant difference between the two cohorts can be explained by the involvement of the nutritionist only in exceptional cases in the SOC, compared to the systematic inclusion in the OGCM. The importance of nutritional counselling is presented by Arkley et al. in relation to a reduction in postoperative complications, through the optimisation of patients’ nutritional status during their hospital stay [31].

Concerning social services costs, higher expenses were incurred with the OGCM treatment approach (*p* < 0.0001), primarily due to the early involvement of social services in the multidisciplinary care model. The increased flow of information between the specialists involved in patient care has made it possible to promote an earlier discharge of patients who have followed the OGCM setting, with a positive impact on direct costs [12,24]. The multidisciplinary nature of the OGCM protocol not only allows patients to be discharged earlier than under the SOC protocol but also to be discharged to the most appropriate facilities and at the most opportune time, considering the feedback obtained on patients’ medical conditions during the biweekly multidisciplinary meetings.

Summarising the previous findings, individual procedures provided following the OGCM protocol generated higher daily costs compared to a SOC setting, except for patient surveillance. However, by taking into account the overall costs generated during the entire hospital stay, the multidisciplinary care approach was not found to be more expensive than the SOC. This result stems from a set of standard orders that enhance the efficiency of patient care, leading to shorter hospital stays. According to Tan et al., this is a key factor that significantly impacts hospital costs [22].

One of the strengths of the present study is the investigation of two well-established care models, with directly comparable real costs, not influenced by the learning effects of newly implemented protocols or the presence of a historical control group [1,14,32]. Furthermore, in contrast to most economic studies, no costs derived from registers or insurance claim data were used, but economic data obtained from the hospital’s finance department were analysed [13,17,23].

Despite the economic results supporting the OGCM protocol, some limitations must be considered. Firstly, our study was not a randomised controlled trial, although patients were admitted to the group following OGCM or SOC depending on the bed availability in the two analysed sites. Secondly, the sample size was not large enough to identify several significant differences in costs between the two cohorts. Nevertheless, clear trends could be identified supported by the robustness of the available data. Thirdly, it was not possible to carry out a cost-effectiveness analysis by means of quality-of-life indicators, as no data were available to perform this type of evaluation. Despite this limitation, the promising clinical and economic results suggest the potential benefits of a multidisciplinary approach.

## 5. Conclusions

The increasing number of proximal femoral fractures in the elderly population represents a serious problem for the finances of the healthcare system. The results of this study highlight how a multidisciplinary care approach can effectively address these challenges without incurring higher costs, thanks to a more efficient allocation of available resources. Considering the supportive and favourable clinical outcomes, our findings suggest that an OGCM protocol can be cost-effective. These findings may support the decisions of policymakers in planning the future healthcare system, with the aim of providing high-quality care in an economically sustainable manner.

## Figures and Tables

**Table 1:** Baseline and demographic characteristics.

Characteristic	OGCM (n = 143)	SOC (n = 141)	*p*-Value
Age [years], mean (±SD)	87.3 (5.6)	86.5 (6.2)	0.25
Women, n (%)	112 (78.3)	99 (70.2)	0.15
BMI [kg/m^2^], mean (±SD)	23.1 (4.2)	23.9 (5.7)	0.22
Fracture type, n (%)			
Trochanteric	72 (50.3)	79 (56)	0.40
Femoral neck	71 (49.7)	62 (44)	0.40
Left side fractures, n (%)	69 (48.3)	55 (39)	0.15
Residence before admission, n (%)			
Home	84 (58.7)	86 (61)	0.79
Retirement home	14 (9.8)	27 (19.1)	0.038
Nursing home	45 (31.5)	27 (19.1)	0.025
Other hospital	0 (0)	1 (0.8)	0.99
Mobility before admission, n (%)			
Independent	55 (39.3)	50 (35.7)	0.62
Stick	19 (13.6)	21 (15)	0.86
Rollator	49 (35)	55 (39.3)	0.54
Walker	0 (0)	1 (0.7)	1
Helping person	4 (2.8)	2 (1.4)	0.68
Wheelchair	13 (9.3)	11 (7.9)	0.83
Barthel Index [0–100], mean (±SD)	30 (17.5)	45.9 (20)	<0.0001
SOMC Test [0–28], mean (±SD)	13.5 (10.1)	9.7 (7.4)	0.018
NRS [0–7], mean (±SD)	3.4 (1)	3 (1.4)	0.06
ASA classification [1–6], n (%)			
2	22 (15.4)	33 (23.4)	0.12
3	108 (75.5)	96 (68.1)	0.21
4	13 (9.1)	12 (8.5)	1
Antithrombotic agents, n (%)	75 (52.4)	81 (57.4)	0.47
Antiosteoporotic medication, n (%)	87 (60.8)	63 (44.7)	0.009

SD = standard deviation. n = number of patients. SOC = standard of care. OGCM = orthogeriatric co-management. BMI = body mass index. SOMC = Short Orientation–Memory–Concentration. NRS = nutritional risk screening. ASA = American Society of Anaesthesiologists Classification. Antithrombotic agents included platelet aggregation inhibitors and antithrombotic medications. Barthel Index, SOMC, and NRS were not systematically collected in the SOC. *p*-value < 0.05 was considered statistically significant.

**Table 2:** Outcome measures.

Outcome	OGCM (n = 143)	SOC (n = 141)	*p*-Value
Length of stay [days], median (IQR)	6 (5–8)	7 (6–9)	0.002
Time to surgery [h], median (IQR)	15.8 (7–26)	15.4 (10–23)	0.77
Readmissions, n (%)			
30 days	40 (35.7)	41 (38.7)	0.40
1 year	9 (6.1)	21 (14.7)	0.76
Revisions, n (%)			
30 days	3 (2.4)	3 (2.4)	1
1 year	9 (8.7)	11 (11.5)	0.67
Mortality, n (%)			
30 days	12 (8.8)	11 (8)	1
1 year	33 (24.1)	43 (31.4)	0.23
Complications, n (%)			
Medical	78 (54.5)	92 (65.2)	0.09
Surgical	9 (6.3)	14 (9.9)	0.37
Delirium, n (%)	54 (37.8)	66 (46.8)	0.16
Residence after discharge, n (%)			
Home	3 (2.1)	4 (2.8)	0.99
Retirement home	9 (6.3)	21 (14.9)	0.030
Nursing home	47 (32.9)	29 (20.6)	0.027
Geriatric rehabilitation	45 (31.5)	65 (46.1)	0.013
Musculoskeletal rehabilitation	33 (23.1)	18 (12.7)	0.035

IQR = interquartile range. n = number of patients. SOC = standard of care. OGCM = orthogeriatric co-management. Medical complications included delirium, cardiovascular, pulmonary, and renal diagnosis, urinary tract infections (UTIs), and bleeding anaemia occurring in the hospital. Data on surgical complications were collected up to 1 year post-surgery. *p*-value < 0.05 was considered statistically significant.

## Data Availability

The datasets used and/or analysed during the current study are available from the corresponding author upon reasonable request.

## Abbreviations

OGCM	Orthogeriatric co-management
SOC	Standard of care
LOS	Length of stay
CHF	Confoederatio Helvetica Franc
USD	United States dollar
ICU	Intensive care unit
IQR	Interquartile range
